# Tuberculous Spondylodiscitis with Psoas Abscess Descending into the Anterior Femoral Compartment Identified Using 2-deoxy-2-[18F]fluoroglucose Positron Emission Tomography Computed Tomography

**DOI:** 10.3390/diagnostics14101018

**Published:** 2024-05-15

**Authors:** Julian Scherer, Tessa Kotze, Zintle Mdiza, Andrew Lawson, Michael Held, Friedrich Thienemann

**Affiliations:** 1 Orthopaedic Research Unit, Division of Orthopaedic Surgery, Department of Surgery, Faculty of Health Science, University of Cape Town, Cape Town 7925, South Africa; 2 Department of Traumatology, University Hospital Zurich, University of Zurich, 8091 Zurich, Switzerland; 3 General Medicine & Global Health Research Unit, Department of Medicine and Cape Heart Institute, Faculty of Health Science, University of Cape Town, Cape Town 7925, South Africa; 4 CUBIC, PET/CT, Department of Medicine, University of Cape Town, Cape Town 7925, South Africa; 5 Department of Internal Medicine, University Hospital Zurich, University of Zurich, 8091 Zurich, Switzerland

**Keywords:** tuberculous spondylodiscitis, Pott’s disease, drug-sensitive, PET/CT, psoas abscess

## Abstract

A 24-year-old immunocompetent woman underwent whole-body 18F-FDG PET/CT for the evaluation of MRI-suspicious tuberculous spinal lesions. The PET/CT results showed no pathological uptake in either lung, and there were no pathological changes on CT. There was increased uptake in the right psoas muscle, extending continuously down anterior to the right hip joint, posterior to and around the trochanteric region of the right femur, and into the right thigh, with an SUVmaxbw of 17.0. Subsequently, the patient underwent CT-guided biopsy as per protocol, which revealed drug-sensitive Mycobacterium tuberculosis, and the patient was started on standard tuberculosis treatment for 12 months.

Spinal tuberculosis usually results in local pain, neurological deficit, spinal instability and fever, and the duration from initial symptoms to adequate diagnosis can be up to several years [1,2,3]. Magnetic resonance imaging (MRI) has a reported specificity of 93% and a sensitivity of 94% in detecting spondylodiscitis and therefore is the current gold standard imaging modality for spinal TB [4]. If possible, an MRI of the whole spine should be performed to detect noncontinuous lesions, which occur in 15 to 20% of the patients [5]. PET/CT has recently been shown to be able to detect (extraspinal) sites of infection and monitor treatment success and has shown greater specificity in the diagnosis of spinal tuberculosis than MRI [6,7]. This case illustrates that PET/CT shows promise as a valuable imaging modality for the initial assessment of spinal tuberculosis and its extraspinal manifestation (Figure 1).

## Figures and Tables

**Figure 1:**
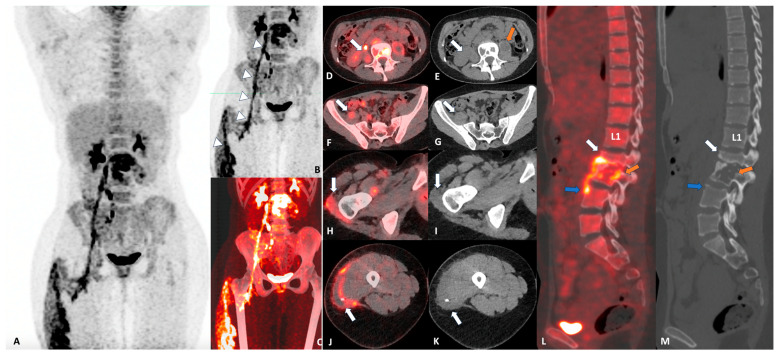
A 24-year-old immunocompetent woman with a history of four months of lower back pain and magnetic resonance imaging (MRI) features suggestive of tuberculous spondylodiscitis of lumbar vertebra 2 and 3 was recruited to the Spinal TB X cohort at the Groote Schuur Hospital of the University of Cape Town (ClinicalTrials.gov Identifier: NCT05610098). As per study protocol, the patient underwent whole-body 2-deoxy-2-[18F]fluoroglucose (FDG) positron emission tomography (PET) computed tomography (PET/CT). PET/CT showed no pathological uptake in either lun’s and there were no pathological changes on CT (**A**). There was increased uptake in the right psoas muscle extending down anterior to the right hip joint, posterior to and around the trochanteric region of the right femur and into the right thigh, with an SUVmaxbw of 17.0 ((**B**,**C**) white triangles; (**D**,**F**,**H**,**J**) white arrows). On CT, the psoas muscle had an altered density, which spread caudally all the way into the lateral compartment of the thigh ((**E**,**G**,**I**,**K**), white arrows). Increased uptake was also seen in the left psoas muscle, with an SUVmaxbw of 8.04. A discrete 80 mm long by 40 mm wide psoas collection is noted on the left on CT ((**E**), orange arrow). In the L2–4 region, vertebral collapse with 50% destruction of L2 ((**L**,**M**), white arrow), significant body destruction of L3 ((**L**,**M**), orange arrow), and beginning erosion of the anterior body of L4 ((**L**,**M**), blue arrow), with an SUVmaxbw of 13, were noted. Subsequently, the patient underwent CT-guided biopsy as per protocol, which revealed drug-sensitive Mycobacterium tuberculosis, and the patient was started on standard tuberculosis treatment for 12 months.

## Data Availability

DICOM data are available from the corresponding author upon reasonable request.
